# Contrasting “back home” and “here”: how Northeast African migrant women perceive and experience health during pregnancy and postpartum in Canada

**DOI:** 10.1186/s12939-016-0369-x

**Published:** 2016-05-25

**Authors:** Maira Quintanilha, Maria J. Mayan, Jessica Thompson, Rhonda C. Bell

**Affiliations:** Department of Agricultural, Food & Nutritional Science, University of Alberta, 4-112 Li Ka Shing Centre for Health Research Innovation, Edmonton, AB T6G 2E1 Canada; Faculty of Extension, University of Alberta, 2-281 Enterprise Square, 10230 Jasper Avenue, Edmonton, AB T5J 4P6 Canada; Department of Agricultural, Food & Nutritional Science, University of Alberta, 4-126 Li Ka Shing Centre for Health Research Innovation, Edmonton, AB T6G 2E1 Canada

**Keywords:** Migrant women, Pregnancy, Postpartum, Health, Qualitative, Focus groups, Focused ethnography

## Abstract

**Background:**

International migration and the number of migrant women who experience pregnancy and childbirth in receiving countries have significantly increased in the last two decades. Migrant women often have unmet social and economic needs during pregnancy, and are more likely to have problems unaddressed by health care systems. In this qualitative study, we explored migrant women’s perceptions and experiences of health during pregnancy and postpartum, while participating in a perinatal program offered through a community-based organization. Additionally, we examined sociocultural factors that might have shaped women’s health upon migration to the Canadian city of Edmonton, Alberta.

**Methods:**

A community-based participatory research approach was used to engage migrant women connected to a community-based perinatal program in Edmonton. A focused ethnography was conducted with four Northeast African communities (Eritrean, Ethiopian, Oromo and Somali), and involved 10 focus groups with women (*n* = 8, per group) and direct observations of weekly perinatal program activities. Data generation and analysis occurred concurrently, and all generated data were analyzed using qualitative content analysis to inductively derive codes and categories.

**Results:**

Women expressed their perceptions and experiences of health during pregnancy and postpartum by contrasting their countries of origin with Canada, respectively identified as “back home” and “here”. Differences in social support and the physical environment (both natural and built) between “back home” and “here” were commonly described as factors that shaped their opportunities to eat healthy, be physically active and emotionally well before and after having a baby “here”. Overall, women described that in Canada they lacked the social and environmental factors perceived as key enablers of healthy pregnancies and postpartum.

**Conclusion:**

A complex network of factors seem to influence Northeast African women’s health during pregnancy and postpartum upon migration to Canada. It is of the utmost importance to provide these women with the immediate sociocultural and environmental factors they need to successfully thrive during pregnancy and postpartum, especially while establishing social and support networks “here”.

## Background

International migration—defined as “a change of residence involving the spatial movement of persons across country borders” [1]—has significantly increased in the last two decades [2]. United Nations data indicates that in 2015 there were approximately 244 million migrants worldwide, with slightly less than half being women [2]. As a result, the number of migrant women who experience pregnancy and childbirth in receiving countries has also increased, sparking the dialogue about the impact of migration on maternal and child health [3–5].

In Canada, the industrialized country with the highest immigrant population as a proportion of the total population among the former Group of Eight (G8) nations [6, 7], low socioeconomic status is more common among migrant families. Immigrant women often have unmet social, economic and health needs during pregnancy, and poorer birth outcomes [8]. A study of migrant women (including refugees, asylum seekers, undocumented and economic immigrants) living in two Canadian cities that receive the highest number of migrants has shown that migrant women are more than twice as likely as Canadian-born women to have problems unaddressed by the health care system [5]. This could be related to the fact that refugees, asylum seekers and undocumented immigrants (i.e., individuals lacking proper visa documentation to reside in Canada) are entitled to fewer benefits and services, and might fear jeopardizing settlement in Canada by accessing health and social services [5, 9].

In addition, women’s cultural background plays a role in reproductive health, and this represents one of the reasons why Mendez, Hogan, and Culhane (2014) stress the importance of considering the intricate interplay of genetic, physiological, behavioural, environmental, and social factors when investigating disparities in perinatal outcomes [10]. A qualitative study conducted with resettled Somali women has shown that even though they appreciate prenatal care in the receiving country, women struggle with language barriers, accessing interpreters, and trusting caregivers who do not have experience in dealing with childbirth among females who have undergone genital mutilation [4]. These sociocultural factors have been suggested as possible explanations as to why rates of gestational diabetes, cesarean delivery and fetal distress are commonly higher among Somali women [11]. The World Health Organization ‘Recommendations on Health Promotion Interventions for Maternal and Newborn Health’ emphasize that cultural factors can affect women’s use of care during pregnancy, and urged for meaningful inclusion of their cultural preferences in quality maternity services [12].

In the Canadian city of Edmonton, Alberta, migrant women may receive additional perinatal support from a community-based organization, the Multicultural Health Brokers (MCHB) Cooperative. The MCHB Cooperative is an independently run health broker cooperative that provides perinatal programs and services to at-risk migrant women, including refugees and asylum-seekers. Health brokers offer women holistic, strategic services that extend beyond a single health concern, such as low birth weight or premature birth. Services include actions related to overarching social determinants of health, such as housing, income, food security and women’s education [13]. The overall goal of health brokers is to promote migrant women’s health during pregnancy and improve birth outcomes by contributing to their successful settlement, adaptation, and integration into Canadian society.

Given all the adaptation that migration requires, in particular during the perinatal period, we sought to explore migrant women’s perceptions and experiences of health during pregnancy and postpartum in Canada while participating in a perinatal program offered through the MCHB Cooperative, and receiving support from a health broker. By exploring women’s perceptions and experiences, we also examined sociocultural factors that can shape their health upon migration.

## Methods

A Community-Based Participatory Research (CBPR) approach was used to engage health brokers and migrant women who participated in perinatal program offered through the MCHB Cooperative in this qualitative study. The project was introduced to all health brokers and four health brokers representing Northeast African communities—Eritrean, Ethiopian, Oromo and Somali—expressed interest in participating in the project to better understand the sociocultural context their clients experience during pregnancy and postpartum in Canada. CBPR was identified as a viable approach for working with these minority groups, and addressing perinatal health disparities that affect migrant women [14, 15].

Focused ethnography was the qualitative research method used in this study. This method is sensitive to how culture shapes and possibly explains women’s everyday lives and perinatal health behaviours [16–18]. Parallel to traditional ethnographic research, in focused ethnography the attention to culture remains; however, it is more contained to a particular setting or focused on certain issues, and within a shorter time frame [16, 18]. Given that our focused ethnography was centered on pregnancy and postpartum experiences, in a contained context (i.e., four Northeast African communities connected to MCHB) and within a shorter time frame, it required prior familiarity with women’s communities [16]. Agar (1996) has described that “devotion to the initial learning role is one of the major ingredients that makes ethnography the unique concoction it is” (p. 120), and this “devotion” was pertinent to our focused ethnography [19]. The CBPR approach enabled us to develop relationships with health brokers, gain background knowledge about Northeast African migrant women’s community contexts in Edmonton where they experience pregnancy, and work with both health brokers and women throughout all stages of the study [20]. Health brokers directed us on the most appropriate data generation strategies, questions to pose to participating women within their communities, and assisted with data interpretation [15, 20, 21].

### Participants and setting

All pregnant and postpartum women from the four participating Northeast African communities who were enrolled in the MCHB perinatal program and attended perinatal activities at the time data were being generated for this project (May-September 2014) were invited to participate. The program involved weekly meetings in which a variety of topics—such as brain and fetal development, healthy eating during pregnancy, labour and delivery—were addressed by health brokers and/or invited health professionals. In addition, participating women had the opportunity to socialize as they shared a meal prepared by the health brokers. This social time also enabled health brokers to distribute resources (prenatal vitamins, donated infant items), and to assist immigrant and refugee women with matters related to housing, education, immigration, food insecurity, income and any other needs they had.

Data generation took place at the MCHB Cooperative office or in another community setting (i.e., school kitchen) during weekly meetings. Health brokers were responsible for explaining the purpose of the research and potential risks and benefits of study participation in women’s mother tongue. They also highlighted that women’s participation was voluntary, and not associated with their enrolment in MCHB programs and services.

Women received a twenty-five-dollar gift card to a local grocery store as an honorarium for focus group participation. Over the course of the study, however, the number of participants increased and the amount provided had to be decreased to 10 dollars per participant per focus group due to budgetary restrictions. This unanticipated protocol change was likely related to the fact that we were working with migrant women who were low income and valued the monetary honorarium, as well as the opportunity to socialize with other members of their communities.

### Ethics

This study received approval from the University of Alberta Research Ethics Board. Due to the language barriers between researchers and participants, interpreters were asked to explain the purpose of the research at the beginning of each data generation point, and reiterate with women that participation was voluntary. Participants gave consent at each data generation point.

### Data generation

Focus groups were conducted in women’s mother tongue to explore their perceptions and experiences of health during pregnancy and postpartum. Focus groups have been described as a particularly useful data generation strategy in health services research with minority groups “whose voices have been otherwise muted” [22], which was highly desirable in our study. Women and health brokers spoke diverse languages and dialects; therefore, the composition of and approach to each cross-lingual focus group varied, with health brokers actively participating either as real-time interpreter (Eritrean, Ethiopian, Oromo) or bilingual moderator (Somali). The different approaches used in these focus group discussions, and format of data generated have been detailed elsewhere [21].

In total, 10 focus groups (approximately 8 participants per group) were conducted with Northeast African migrants who had been living in Canada between 1 and 36 months: 3 with Ethiopian and Eritrean women, 3 with Oromo women, and 4 with Somali women. After each focus group discussion, we (i.e., the research team) reviewed what questions had been explored with women, and what questions or topic areas had yet to be examined with each participating community. We engaged health brokers in these discussions as a key principle of CBPR, and in an effort to better understand women’s cultural background, social context, and preferred ways of sharing their stories. We have described the important role of health brokers in an earlier publication [21].

In addition to focus group discussions and time spent with health brokers, we also interacted with women by observing weekly perinatal classes that addressed a variety of topics (e.g., brain and fetal development, delivery, healthy eating, etc.). As such, focus group data were supplemented with data from direct observations. Data from direct observations were documented in audio-recorded debriefings that occurred between the two researchers responsible for all data generation (MQ and JT).

### Data analysis

Data generation and initial stages of data analysis occurred concurrently, and required an inductive, iterative, self-reflective analytic process [17]. Audio recordings of focus groups and debriefings were transcribed verbatim, and organized in NVivo (Version 10.0.4, QSR International). Transcripts of focus groups and debriefings were reviewed for accuracy, and organized so that field notes were added to transcribed documents in the form of highlighted comments in a word document. All data were analyzed using qualitative content analysis to inductively derive codes and categories [23, 24]. In this focused ethnography, qualitative content analysis was referred to as “a research method for the subjective interpretation of the content of the text data through the systematic classification process of coding and identifying themes or patterns” [25]. While analyzing focus group data, we were also sensitive to interactions among participants, and between participants and health brokers [21]. Transcripts from all data sources were read multiple times, and initial codes began to be identified. These codes were reduced, and grouped into key categories and sub-categories that were critically interpreted by the research team to provide a rich description of findings. Therefore, findings presented here were derived from focus groups and observational data.

## Results

We opened the first focus group discussion with women from Ethiopian, Eritrean, Oromo and Somali communities with a general question of what it meant for them to be healthy during pregnancy and postpartum. Women then began to express their perceptions and experiences of health during pregnancy and postpartum by contrasting their countries of origin with Canada, which were described as “back home” and “here”, respectively. As a result, in the following focus group discussions, we explored how differences between “back home” and “here” might have shaped their health before and after having a baby. Women commonly identified eating healthy, being physically active and emotionally well as pivotal aspects of a healthy pregnancy. We will describe here how participants perceived social support and the physical environment (both natural and built) “back home” and “here” as key factors in shaping their perception of health during pregnancy and postpartum in their new country, Canada.

### Social support

Participants described that in their home countries pregnancy and childbirth were highly valued by society regardless of women’s socioeconomic status. As such, pregnant and postpartum women were treated as “queens” who had their cravings filled and needs tended to by female relatives, friends or hired help. “Back home” pregnancy was a time when women received the most attention from their families, including their spouses, which was something they truly appreciated and still expected after moving to Canada. In addition, Northeast African migrant women emphasized that, despite political and civil conflicts in their home countries, they were part of a “we culture” within their communities (e.g., neighbourhoods, villages, families) where individuals cared for one another’s needs and well-being. This was heightened when a woman got pregnant, and the support and attention she received during pregnancy and postpartum were perceived as critical to her overall health as well as emotional well-being:

*“After birth, women get all the emotional support from all extended family members. There are always people surrounding you. You get a break from the baby and are encouraged to work or do different things.” (Summary of Somali women’s focus groups)*

Social support “back home” was commonly linked to women’s ability to eat healthy and be physically active, two health behaviours participants identified as key during the perinatal period. As examples, women highlighted that people in their communities would prepare foods they were craving or were special in their cultures (e.g., lamb, dishes associated with healing), and cook for them when they did not feel well or were recovering from childbirth. In relation to physical activity, women believed social support from their relatives and friends “back home” was a facilitator to being physically active, as described in the quote below:

*“So you see in our country even if you are poor there is always someone who can help you, it could be a relative, it could be someone you even pay. And when they do that you are out relaxing, going for a walk, you know doing things that you would like (…)”* (*Ethiopian woman, mother of two)*

In contrast to participants’ perception of social support “back home” during pregnancy and postpartum, “here”, in Canada, women commonly experienced a more individualistic culture, described as an “I culture”, where every woman is held responsible for her own health and well-being. They noted that family units were usually smaller “here”, and felt that Canadian men (i.e., women’s partners or spouses) tended to have a more active role in looking after children and helping with house chores. Although women perceived the difference in male’s roles between “back home and here” to be positive, it was not what they personally experienced within their households in Canada since their spouses did not naturally begin to share housework with them upon moving “here”.

Living in an “I culture” without extended family members and friends’ support made women feel they lacked the emotional and instrumental (e.g., financial support, hired help) resources to eat healthy and be physically active while pregnant and postpartum. For instance, women shared that they were aware of healthy eating “here” but the cost of healthy foods commonly prevented them from eating what they believed they should. If they were in a similar situation “back home”, it was likely someone in their communities with more resources would share what they had to help them to eat healthy. This was captured in audio-recorded direct observations of programming where researchers observed a participant pointing to another woman around the table while saying that if that woman was rich, and they were living back home, then a quarter of what she had would be shared with the community, “Here”, on the other hand, they felt they had to live and deal with scarce resources on their own:

“*Here, in Canada, even doctors and nurses don’t really consider poverty, they just kind of treat everybody the same, you go in, they give you a list of things that you need to do but they don’t really consider what it’s like living inside and outside our daily lives.” (Oromo woman, mother of one)*

Overall, women commonly described lack of social support as a factor that negatively shaped their pregnancy and postpartum experiences “here”. They believed that their lives in Canada, in the absence of kin, hindered their opportunities to eat healthy and be physically active, increased their stress, and decreased their emotional well-being. In fact, some women underlined that the experiences they faced “here” clashed with their expectations that Canada would be a “flexible, easy place to live”, and made them question whether their lives “here” were really better than in the midst of political conflicts “back home”.

### Physical environment

Participants also perceived that differences in the environment, both natural and built, between “back home” and “here” played a role in their eating habits and physical activity during pregnancy and postpartum. Interestingly, eating healthy “back home” was commonly described as consuming “organic” foods that were available to women throughout the year. In this case, “organic” encompassed fresh, unprocessed foods that were either farmed locally or procured on a daily basis at the local market. Although food variety was not extensive, the tropical weather provided a favourable climate for growing vegetables, fruit, and grains in home gardens for families’ subsistence. “Here”, on the other hand, the summer season only gave women a short time frame to grow and eat locally grown vegetables and fruit, and not all of them had a place to cultivate a garden.

Furthermore, participants’ natural environment “back home” was also more conducive to being physically active as the weather was warm throughout the year—*“we come from 13 months of sunshine*”—and enabled farming and walking to the market daily. In contrast, women discussed how the natural environment in Canada placed some barriers for them to be physically active. The weather “here” was a limiting factor for how much physical activity they were able to do during the winter. During winter women usually took the bus or drove everywhere, and they lacked the time and financial resources to enroll in indoor fitness classes and gyms that suited their needs.

Although “weather” was a very common theme in the discussion of environmental differences between “back home” and “here”, women also considered the fact that the built environment “here” offered an abundance of unhealthy, convenience foods that could be bought or ordered for take out when they did not feel like making homemade meals. High availability of low-cost unhealthy foods while coping with poverty, extensive hours of work during pregnancy, low social support, lack of sleep and postpartum depression were given as reasons why women resorted to unhealthy convenience foods even though they understood it was not the best choice for their health:

*“Here if you are busy, if you have to run around, if you have to work, and you have to do house chores you might not get enough sleep. And you might have to cook but you don’t want to cook because you are tired so women ask their husbands to buy food from the restaurant while he is coming to home.” (Eritrean woman, mother of two)*

Overall, participants’ perceived the physical environment and social support “back home” as enabling factors for healthy pregnancies; whereas “here” they missed those factors in their daily lives, and felt unprepared to deal with their new realities as discussed by Somali women, *“these are new terms to our life, and we have no cultural background to address [them]”*.

It is worth noting that participants perceived services and supports offered through MCHB Cooperative (e.g., childcare, cooking classes, family activities) as facilitators to their health and emotional well-being during pregnancy and postpartum in Canada. Women commonly described health brokers with whom they were connected through the MCHB Cooperative as individuals they relied on for guidance and support throughout pregnancy and after delivery. Additionally, MCHB perinatal program fostered social interactions among women from African communities, thus mimicking some of the sense of community women experienced “back home”. This seemed to help creating a sense of belonging “here.”

## Discussion

Northeast African migrant women recognized the importance of healthy eating, and being physically active and emotionally well for a healthy pregnancy and postpartum. However, this was mostly done as women contrasted the social support and physical environment “back home” and “here”, and explained how these differences have shaped their experiences while pregnant and postpartum in Canada.

The definition of social support includes “resources and aid derived from one’s social relationships” [26]. The types of support widely cited in the literature investigating the relationship between social support and pregnancy outcomes are emotional and instrumental (which may include informational support) [26, 27]. Emotional support refers to relationships that make one feel loved, cared for and appreciated, whereas instrumental support includes tangible assistance that answers specific needs, such as lending money, childcare, offering housing or something as simple as a ride to the hospital [26]. It is evident that “back home” women were able to easily access emotional and instrumental support in pregnancy and postpartum through their families and communities, in particular female relatives and friends. This enabled them to carry healthy behaviours during and after pregnancy, and also made them feel cherished, like “queens.” Northeast African migrant women missed the collective support in pregnancy and early postpartum in Canada, which seemed to negatively affect their experiences “here.”

Qureshi and Pacquiao (2013) described similar findings among migrant women of Pakistani origin living in New Jersey (US) who described missing the comfort, care, emotional support, and teachings about marriage and birthing they would receive while pregnant or postpartum in Pakistan [28]. Interestingly, 8 of their 26 participants postponed pregnancy for between 1 and 5 years post migration. Those who did so were able to develop a network of friends in the US, learn about health and social supports that were available to them, and develop a new husband-wife relationship in which household activities were more evenly distributed [28]. Northeast African migrant women who participated in our study had yet to develop a strong social network or experience the transition in the husband-wife relationship, and this could be related to the fact that participating women were new to Canada, having lived “here” for 1 to 36 months. Moreover, the number of immigrants of African origin coming to Canada only began to increase in the last two decades [29]; therefore, women may not be easily welcomed into established communities but would likely need to develop their own community circles.

Pregnant and postpartum Latino women in the US have also linked social support to their motivation and beliefs about the need to stay healthy in pregnancy, and described the absence of their mothers and female relatives as a barrier to eating healthy and exercising [27]. Therefore, interventions to promote maternal health among migrant women may better meet their needs and expectations by following approaches that are community-based and family-oriented while fostering migrants’ social inclusion [5, 27, 30].

In addition to social support, it is important to note the implications of the physical environment “here” on participants’ pregnancy and postpartum health experiences. The high cost of healthy foods and the abundance of low-cost unhealthy foods represented barriers Northeast African migrant women faced to eating healthy before, during pregnancy and after delivery in Canada. Low income and food insecurity have been widely reported among migrant families and ethnic minorities [8, 31–34], with a high proportion of asylum-seeking women reporting skipping meals during pregnancy due to the lack of resources [31]. Household food insecurity may also increase pregnant women’s consumption of foods that are high in fat, sugar and salt as stress-relief mechanism or as strategy to eat in low-cost ways [32, 33, 35]. Participants in our study commonly reported consuming unhealthy foods (e.g., fast foods) as an affordable way to ease and cope with their busy, stressful lives in Canada. This practice did not occur “back home” because fresh foods, produced and accessible locally, were the only options consistently available to them, and if financial resources were scarce they could count on their social networks for help.

Overall, Northeast African migrant women described feeling unprepared to deal with their new lives in Canada that included poverty and isolation. Based on results presented here, participants’ resilience (i.e., a complex process in which psychological, social, environmental factors interact, and make an individual more capable of coping with adversities [5]) might be perceived or interpreted as low. However, we draw from Gagnon and colleagues (2013), and emphasize the fact that resilience among migrant women is a phenomenon resulting from the individual-environment interaction [5]. Our findings show that new Northeast African migrant women do not readily establish their social networks and supports upon migration, and recognize that Canada, as a receiving nation, might not provide them with the sociocultural and environmental factors they need to successfully adapt and thrive “here” in the short term. This highlights the importance of perinatal community-based programs, such as those offered through MCHB Cooperative, which can provide new migrant women with much needed social support.

As such, these programs have the potential to help create some of the sociocultural context that women experienced with pregnancy and postpartum “back home”, and promote social inclusion by giving them opportunities to socialize; develop new social networks; and discuss their realities in Canada. Moreover, the health-brokering model embedded in the MCHB Cooperative program has been able to successfully integrate social and health services with the aim of promoting communities’ health [13, 36]. The integration of social and health services is of the utmost relevance for international migrants who may have limited access to health care services while awaiting for official settlement in the receiving country [31].

This focused ethnographic study provided a rich description of Northeast African migrant women’s perceptions and experiences of health during pregnancy and postpartum in Canada. The CBPR approach allowed researchers and health brokers to foster women’s participation, and better grasp their sociocultural environments “back home” and “here.”

Since this study was a qualitative investigation of women’s perceptions and experiences of health during pregnancy and postpartum, no socio-demographic data were formally collected. Participants were asked at the end of focus groups to share, if comfortable, for how long they had been living in Canada. Although health brokers from Eritrean, Ethiopian, Oromo and Somali communities disclosed that many participating women had refugee status, based on the data generated we were not able to infer that our findings reflected the views of Northeast African migrant women in any specific immigration class (e.g., economic migrant vs. refugee). This could be seen as a limitation of our study since refugee and asylum-seeking women might be more vulnerable to the additional challenges migration (e.g., loss of their social networks), especially while pregnant and postpartum, given that they have to deal with more regulatory restrictions in Canada and may have limited fluency in official languages [8, 31]. Yet, our findings shared great similarities with those of other studies conducted with migrant women from different ethnic backgrounds in other parts of Canada and the world [4, 28, 30, 37]. This strongly suggests that the knowledge gained through this qualitative study provides insights that are relevant to other groups and settings investigating pregnancy and postpartum experiences of migrant women.

## Conclusion

Given the complex network of factors that influence migrant women’s health during pregnancy and postpartum in a receiving country, it is of the utmost importance to support them with strong integration policies [38]. Interventions targeting pregnant and postpartum migrant women need to address key social determinants of health, such as income, social support network and education [5].

Community-based organizations, such as the MCHB Cooperative, that work with Northeast African migrant pregnant and postpartum women have the potential to improve women’s health by providing culturally appropriate prenatal/postnatal programs and services that foster women’s social integration into their new countries, and help them build strong social networks in a timely way. Together this will assist them to be healthier themselves and support the health of their families.

## Abbreviations

CBPR, community-based participatory approach; MCHB, multicultural health brokers.
